# Snakebite care through the first two waves of COVID-19 in West Bengal, India: a qualitative study

**DOI:** 10.1016/j.toxcx.2023.100157

**Published:** 2023-04-12

**Authors:** Soumyadeep Bhaumik, Deepti Beri, Anthony B. Zwi, Jagnoor Jagnoor

**Affiliations:** a The George Institute for Global Health, University of New South Wales, Sydney, Australia; b Injury Division, The George Institute for Global Health, New Delhi, India; c School of Social Sciences, University of New South Wales, Sydney, Australia

**Keywords:** Access to care, COVID-19, Health systems, India, Snakebites, Quality of care

## Abstract

Snakebite is a public health problem in many countries, with India having the highest number of deaths. Not much is known about the effect of the COVID-19 pandemic on snakebite care. We conducted 20 in-depth interviews with those bitten by venomous snakes through the two waves of COVID-19 (March–May 2020; May–November 2021), their caregivers, health care workers and social workers in two areas (Sundarbans and Hooghly) of West Bengal, India. We used a constructivist approach and conducted a thematic analysis. We identified the following themes: 1. Snakebite continued to be recognised as an acute emergency during successive waves of COVID-19; 2. COVID-19 magnified the financial woes of communities with high snakebite burden; 3. The choice of health care provider was driven by multiple factors and consideration of trade-offs, many of which leaned toward use of traditional providers during COVID-19; 4. Rurality, financial and social disadvantage and cultural safety, in and beyond the health system, affected snakebite care; 5. There is strong and shared felt need for multi-faceted community programs on snakebite.

We mapped factors affecting snakebite care in the three-delay model (decision to seek care, reaching appropriate health facility, receiving appropriate care), originally developed for maternal mortality. The result of our study contextualises and brings forth evidence on impact of COVID-19 on snakebite care in West Bengal, India. Multi-faceted community programs, are needed for addressing factors affecting snakebite care, including during disease outbreaks - thus improving health systems resilience. Community programs for increasing formal health service usage, should be accompanied by health systems strengthening, instead of an exclusive focus on awareness against traditional providers.

## Highlights

• COVID-19 influenced snakebite care in a multitude of ways during the different waves of COVID-19 in India.
• The choice of health care provider for snakebite is multi-factorial in origin and not solely determined by tradition.
• Multi-faceted community programs for snakebite can contribute to improving health systems resilience.
• Community programs for increasing formal health service use, should be accompanied by health systems strengthening.

## Graphical abstract

**Figure:**
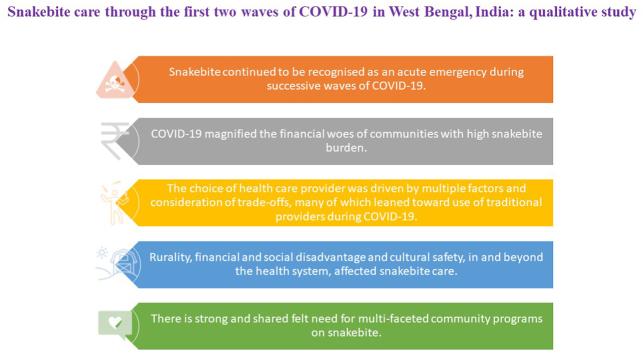

## 1 Background

Snakebite is a significant public health problem in several countries, with India having the highest number of deaths (Chakma et al., 2020; GBD 2019 Snakebite Envenomation Collaborators, 2022; Menon et al., 2017; Suraweera et al., 2020). In 2019, India had the second highest age-standardised mortality rate (4.0 per 100,000), indicative of inadequate snakebite care. (GBD 2019 Snakebite Envenomation Collaborators, 2022).

In May 2019, the World Health Organization (WHO) released a strategy with the explicit target of halving the global burden of snakebite by 2030 (WHO, 2019). A few months after the release of the WHO strategy, on January 30, 2020, COVID-19 was declared as a Public Health Emergency of International Concern by WHO (Eurosurveillance Editorial, 2020). Subsequently, as COVID-19 spread globally, its control through containment measures (social, economic, and mobility-related), together with diversion of scarce health systems resources to scale up the COVID-19 response impacted healthcare delivery. The impact of COVID-19 on care for several conditions has been studied (Cruden et al., 2021; Di Toro et al., 2021; Laura et al., 2021; Lipman et al., 2021; Raman et al., 2021; Rathore et al., 2021; Tandon, 2020), but little is known with respect to snakebite. To the best of our knowledge, only one qualitative study (van Oirschot et al., 2021), has been undertaken in the early phase of the pandemic to understand perceptions of key informants.

We aimed to fill this gap by conducting a qualitative study to explore the effect of COVID-19 on access to appropriate timely care for snakebite envenomation through the two waves of COVID-19 in West Bengal, a state in eastern India.

## 2 Methods

### 2.1 Study context

The Union Government of India, during the initial phase of the COVID-19 pandemic, implemented a complete nationwide lockdown from March 25, 2020 to May 31, 2020. Restrictive measures were gradually eased up until November 2020. These first wave lockdowns were largely successful in containing COVID-19 deaths. However, from March 2021, a surge of COVID-19 cases led to overburdened health systems with unprecedented deaths and suffering due to COVID-19 (Samarasekera, 2021). During the second wave, state governments once again instituted containment measures; in West Bengal these were imposed from May to November 2021.

### 2.2 Study setting

The study was carried out in two geographic areas: semi-rural communities in Hooghly, and rural communities in the Sundarbans of West Bengal, India. Hooghly is known for its high agricultural productivity and proximity to the National Highway which enables connectivity to tertiary health facilities in Kolkata, the state capital. The Sundarbans is a deltaic region and is one of the poorer districts in the state. Transport connectivity is not well developed, and the area is largely rural. The two study areas were chosen purposively noting the difference in terms of degree of rurality and accessibility.

In the first wave of COVID-19, both study areas were under lockdown in accordance with the policies for Union Government of India. The Government of West Bengal, in recognition of the difficulty in managing COVID-19, should an outbreak occur, took more strenuous preventive measures (awareness and isolation) in Sundarbans. Further, there were large scale evacuations due to Cyclone Amphan in May 2020 and Cyclone Yaas in May 2021, that coincided with COVID-19 waves, which led to large scale destruction of infrastructure, loss of livelihoods, and rendering physical distancing near-impossible. In comparison, the Hooghly district had relatively less spread, and COVID-19 was not compounded with natural disasters. In both the places, there were more cases of COVID-19 in the second wave, than first.

### 2.3 Methodological orientation and theory

We used a constructivist approach. Constructivism (Mills et al., 2006) allowed us to emphasise how participants constructed their reality and simultaneously acknowledge the subjective nature of its interpretation during analysis.

### 2.4 Participant selection

We conducted maximum variation purposive sampling, based on study areas and the timing in which a person was bitten (first lockdown in 2020, second lockdown in 2021, and when no lockdown measures were in place). We conducted in-depth interviews with adult participants in Sundarbans and Hooghly, who were survivors or caregivers of venomous snakebite, and were either bitten when COVID-19 containment (lockdown) measures were in place (first and second waves) or when they were lifted (period between two waves or after second wave) irrespective of hospitalisation, and with healthcare and social workers involved in snakebite care. We excluded those with diagnosed cognitive/mental impairment and those not able to provide informed consent. We also excluded participants who were bitten by snakes after February 2022. We disseminated information about the study to potential participants with the help of local organisations. The interviews were all conducted at the homes of survivors, caregivers, and social workers. Healthcare workers were interviewed at their home or at health facility, based on their preference. Interviews were conducted in the absence of non-participants.

### 2.5 Data collection

A semi-structured topic guide, iteratively revised as the study progressed, was used for in-depth interviews (IDIs) in Bangla and English (only one). No particular order of questioning was followed, allowing emphasis on the flow of conversation. The IDIs lasted 14–65 min. The IDIs were audio recorded, with supplementary field notes taken. We interviewed participants on a single occasion.

### 2.6 Analysis

We transcribed IDIs verbatim. Transcripts were not returned to participants. We conducted data collection simultaneously with the process of coding, organising the data and facilitating constant comparison in an iterative and reflective manner. We used thematic analysis. Open coding was done on five transcripts by two authors independently (SB- without any translation & DB on translated transcripts in English). After that, the research team jointly looked for utility and conceptual relations between codes to develop concept maps, which served as the initial coding tree. At this instance codes applied were data-driven, with more interpretive analysis occurring later. This initial coding tree was applied to other transcripts (with no translation by SB). As interviews progressed, the existing coding tree was modified iteratively (in consultation with other authors). The process continued until data saturation was reached for both the study areas separately. The final coding tree was applied to all transcripts. We used NVIVO 11 (Version NVivo Pro). No participant checking was done.

We mapped all factors affecting snakebite care diagrammatically using the three-delay model (originally developed for maternal mortality (Thaddeus and Maine, 1994)): decision to seek care from formal health systems reaching appropriate health facilities and; receiving appropriate care after reaching health facility.

### 2.7 Research team and reflexivity

The research team comprised professionals with backgrounds in medicine, public health, injury research, snakebite, and social work and was gender balanced. All authors had prior experience of qualitative research. The lead researcher (SB) is from West Bengal, an insider. At the same time, considering socio-economic privileges, he is an outsider to the lived realities of the study participants (except for clinicians in the category of health care workers). Others are outsiders. Consistent with a constructivist approach, we worked reflexively, pausing to reflect on any assumptions regarding the interpretation of data, discussing with team members to maintain emphasis on the reality as seen by participants.

## 3 Results

We conducted 20 interviews (Table 1: Summary Characteristics of participants).

**Table 1:** Summary characteristics of participant.

Study areas	- Hooghly: 9 - Sundarbans: 11		
Gender	- Male: 10 - Female: 10 - Other: 0		
Age Group	- 18–30 years: 7 - 31 to 50 years: 11 - > 50 years: 2		
Type of study participant	Snakebite survivors/lay or community caregivers: 10 (survivors 8; lay caregiver 2)	Healthcare worker: 6	Social worker: 4
	• Gender: Male 5: Female −5 • Age: 18-30 years-3; 31-50 years- 5; >50 years: 2 • Profession of person bitten by snake: Housewife −4; Housemaid-1; NGO worker −1; Retired- 1; Migrant worker-1; Farmer-2	• Gender: Male-1; Female-5 • Age: 18-30 years-3: 31-50 years- 3 • Profession: Medical Doctor −1: ASHA-3; Rural Hospital Counsellor-1; Anganwadi −1	• Gender: Male-4 • Age-18-30 years-1: 31-50 years- 3 • Profession: Social workers associated with community-based organisations or community clubs-4

**Fig. 1:**
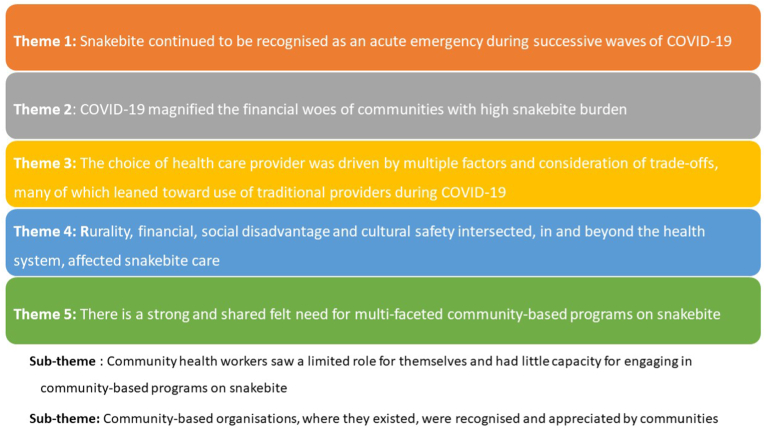
Summary of themes and sub-themes of the study.

Many snakebite survivors and caregivers acknowledged their lack of reference point regarding how snakebite care may have been affected by COVID-19. They described how they navigated a complex set of factors to access snakebite care, including some related to COVID-19. Healthcare workers and social workers on the other hand described many challenges due to COVID-19 containment, over and above the existing challenges in delivering snakebite care. Our analysis of the social understanding of the effect of COVID-19 on snakebite care is presented in the form of five themes, summarised in Fig. 1.

### 3.1 Themes

#### 3.1.1 Theme 1: Snakebite continued to be recognised as an acute emergency during successive waves of COVID-19

Participants recognised that snakebite is an acute medical emergency for which care needs to be sought. This understanding was sustained through the waves of COVID-19. Some participants described fear of contracting COVID-19 as leading to some delay, as they waited for envenoming symptoms to evolve before making the decision to seek care.

*“People were afraid of COVID … Perhaps they thought a bit before going to the hospital, but the effect of COVID is not much (on decision to seek care).”*

*-IDI 009 Snakebite Survivor, Hooghly*

Some participants considered the odds, deciding that the probability of fatality from snakebite was higher than the likely consequences of COVID-19, particularly when taking adequate precautions. The rational frame was more dominant during the second wave of COVID-19 (2021), due to increased confidence and awareness of COVID-19 control measures.

*“There was serious lockdown, but by then we had a mental map … We had a much clearer understanding of what we are facing or what we will be experiencing, what could be the consequences.”*

*– IDI 006 Social*W*orker, Hooghly*

#### 3.1.2 Theme 2: COVID-19 magnified the financial woes of communities with high snakebite burden

Most participants highlighted the poor socio-economic status of snakebite-affected communities and associated an incident of snakebite with inevitable out-of-pocket health care expenditure (costs for transportation and medicines, plus costs and expenses of caregivers while the patient was admitted to a health facility). The financial consequences added to the financial woes of communities with high snakebite burdens due to inflationary pressures.

*“Ambulance, car rental costs a lot to go to the hospital from here.”*

*- IDI 021, Snakebite Survivor, Sundarban*

*“Those who are at the lowest strata of society have a problem. They earn daily and eat daily. They had problems when there were lockdowns. It was long”. – IDI 014, CHW, Sundarban*

A number of social workers and community health workers (CHW) mentioned that COVID-19 had rendered their financial condition more precarious:

*“We do not get a lot of money. Those days we had to bear a lot of pain. … I sell vegetables to make ends meet, but at that time no one had money to buy … The cost of education of children has also increased. It needs a lot of money …. We were dependent on government relief.*^1^*We did not have even money to buy rice and pulses.” - IDI 016, CHW, Sundarban*

We found one community fund in Hooghly which provided financial risk protection for those with snakebite (and all other acute medical conditions), although its sustainability was described as challenging. The fund was accessible at any time, did not need any financial guarantees, and was available for all to meet out-of-pocket expenditure when seeking admission in formal health facilities. The seed for the fund was acquired from the West Bengal Chief Minister's grant-in-aid to community clubs and was replenished from time to time by well-off community members or by snakebite survivors.

#### 3.1.3 Theme 3: The choice of health care provider was driven by multiple factors and consideration of trade-offs, many of which leaned toward use of traditional providers during COVID-19

The choice of healthcare provider involved consideration of multiple factors and trade-offs, this included distance, availability, trust, affordability, and perceptions of outcome.

*“. Rs 100, Rs 50, or Rs 51, whatever we give, Ojha (traditional provider) heals us and is happy with that. That’s why we all go to him. When we went there (hospital), the doctor says it would cost Rs 5000, or Rs 3000, he would write it in the prescription, we would have to bring the medicine, we would have to run around. The ferry does not run at night. How will we return? For traditional providers we pay Rs 10 to the van and we reach home”*

*- IDI 020, Snakebite Survivor, Sundarban*

*“From those who go to hospital, some recover, some are sick, some even die. Everyone who takes the medicine from the Ojha recovers. How will we know (predict) what happens in a hospital?”*

*IDI -013, Snakebite Survivor, Sundarban*

Factors promoting a preference for traditional providers were accentuated during COVID-19.

*“I don't think they had any clue because everyone was so overwhelmed with COVID. If somebody required ventilators, they usually referred to higher facility, and the ventilators were already occupied by COVID patients there. Hospitals in our area, they did not get those extra ventilators … and I don't think they have those extra ventilators now also. So well in rural hospitals, they never have ventilators.” ‘– IDI 006, Social Worker, Hooghly*

A small number of participants mentioned that engagement with members of community-based organisations (CBOs) during the decision-making process moderated the trade-off positively towards accessing formal health systems.

#### 3.1.4 Theme 4: Rurality, financial, social disadvantage, and cultural safety, in and beyond the health system, affected snakebite care

The navigation of snakebite care related to the intersection of rurality, financial and social disadvantage, and perceptions of cultural safety.

In Hooghly, which is semi-urban and connected to the National Highway, geographic access was of relative less concern. Barriers which were financial in nature, related to reaching an appropriate health facility, and receiving appropriate care on reaching the health facility were discussed more. Participants from Sundarbans additionally mentioned distance and availability of transport. Few participants from Sundarbans used an ‘outside land’ framing when discussing referral to tertiary care facilities (usually in urban areas), implying on their lack of familiarity and cultural safety. The lack of cultural safety in accessing care for people in Sundarbans was a challenge to access care for acute medical emergencies, like snakebite.

*“Humans are bitten by snakes everywhere, but we have no hospital here for snakebites. We have to go outside. - IDI 016, Healthcare Worker, Sundarbans*

Some participants expressed those attitudes, behaviours, and communication of medical staff was an influence on care delivery after reaching a formal health facility (in both Hooghly and Sundarban). Participants mentioned that a condescending and unconcerned attitude in emergency departments made health systems navigation difficult.

*“… We entered the emergency office … She (medical doctor) said, you first get the ticket and then do what they say from there. I said, are you crazy? A baby boy is bitten by a snake, and I go outside and get a ticket and then come to you! By then, something serious could happen to the boy. You seem to know a lot, she told me.…Doctors do not behave well in the hospitals. Doctors must be called from their quarters … even if the treatment is started 5 minutes ago, then it increases the probability of their survival. There is a lot of negligence seen. “*

*- IDI 001, Social Worker, Hooghly*

Few participants identified that in first wave, the concerns about COVID-19 in medical staff, might have added to disrespectful behaviour: caregivers were often not communicated about prognosis, or even allowed to enter health facility premises. Health facilities, in which medical staff actively communicated to allay panic and respond to patients and caregiver concerns, were seen to be exceptions. This added to institutional legacy on community preference for specific health facilities for accessing around snakebite care, and many without consideration of distance or time.

*“We counsel them that this is required, and this is not. We allay their panic. In case of any problem, we ask them to call us, and we tell them to come to our hospital.”*

*- IDI 007, Healthcare Worker, Hooghly*

The need for health systems strengthening across all health facilities and learning from best practices of institutions with legacy of good quality of care, was recognised as a need by few participants.

One participant mentioned about caste-based discriminatory behaviour during the first lockdown as a barrier.

*“a lot of general caste people, they look at suspicion, they don't want to help backward people. So, in a couple of cases where there were snakebites, when we were going to respond to the snakebite to evacuate them, take them to the hospital, we were stopped by the police,” – IDI 006, Social Worker, Hooghly*

#### 3.1.5 Theme 5: There is a strong and shared felt need for multi-faceted community-based programs on snakebite

There is a strong and shared felt need for multi-faceted community-based programs on snakebite in high-burden communities. This need was felt in both study areas and expressed by almost all participants either by giving suggestions for improving and scaling up existing activities or by identifying of absence of community-based programs as a gap. Many social workers and patients/caregivers identified with the values of snake conservation, and the need to be non-violent towards “helpless” animals.

Participants identified the following facets of a community-based program.

• awareness on snakes, snakebite prevention and post-bite dos and don'ts
• mitigation of snake-human-environment conflict, including but not limited to ‘snake-rescue’ (translocation of snakes) and promotion of snake conservation
• first-aid and bystander training
• promotion of the use of the formal health system through snake identification, support for decision making on care-seeking, establishing contact, and arranging transport; support during referral to higher centres, and providing advance information to providers in health facilities to ensure preparedness on arrival
• advocacy for strengthening health systems capacity for snakebite care

**Sub-theme**: *Community health workers saw limited role for themselves and had little capacity for engaging in community-based programs on snakebite*.

For many participants, the role of CHWs (Accredited Social Health Activists or ASHAs) in community-based program on snakebite was seen to be limited. CHWs aligned their identity to working for reproductive, maternal, neonatal and child health services. CHWs were overburdened and COVID-19 related services (and extreme weather events, like cyclones, in Sundarbans) added to the challenge.

*“As an ASHA, our work is mainly on maternal and child health – that was how we started initially. Not only do we take care of mothers and children but over and above, additional jobs are thrust on us. Our workload continues to increase every passing day.” -IDI 008, Healthcare worker, Hooghly*

**Sub-theme**: Community-based organisations (CBO), where they existed, were recognised, and appreciated by communities.

The CBOs, where they exist, and although challenged during COVID-19, were recognised, and appreciated by communities. Healthcare workers acknowledged support from CBOs and appreciated their capacity, while some survivors acknowledged their role in advocacy for health systems strengthening.

*“CBOs explain (that) it is not God, but a human who earns profits in the name of cure. The Canning Juktibadi (Science Rationalist) Organisation have capacity to convince people”- IDI 014, Healthcare*W*orker, Sundarbans*

*“… if there is awareness, by CBOs it will be good. I do not think a government can do this, both need to work collaboratively to raise awareness – village by village, intensively through Jatras (folk theatre) then people will benefit” -IDI 019, Snakebite Survivor, Sundarbans*

Through multiple interviews, some inherent advantages, of CBOs in delivering community-based programs was evident: being embedded and always accessible to community, appreciation of cultural and social processes, capacity for snake identification, translocation of snakes (snake-rescue), perceived selflessness, and trust. Lack of recognition and resource constraints were identified as challenges.

### 3.2 Summary of factors affecting snakebite care

**Fig. 2:**
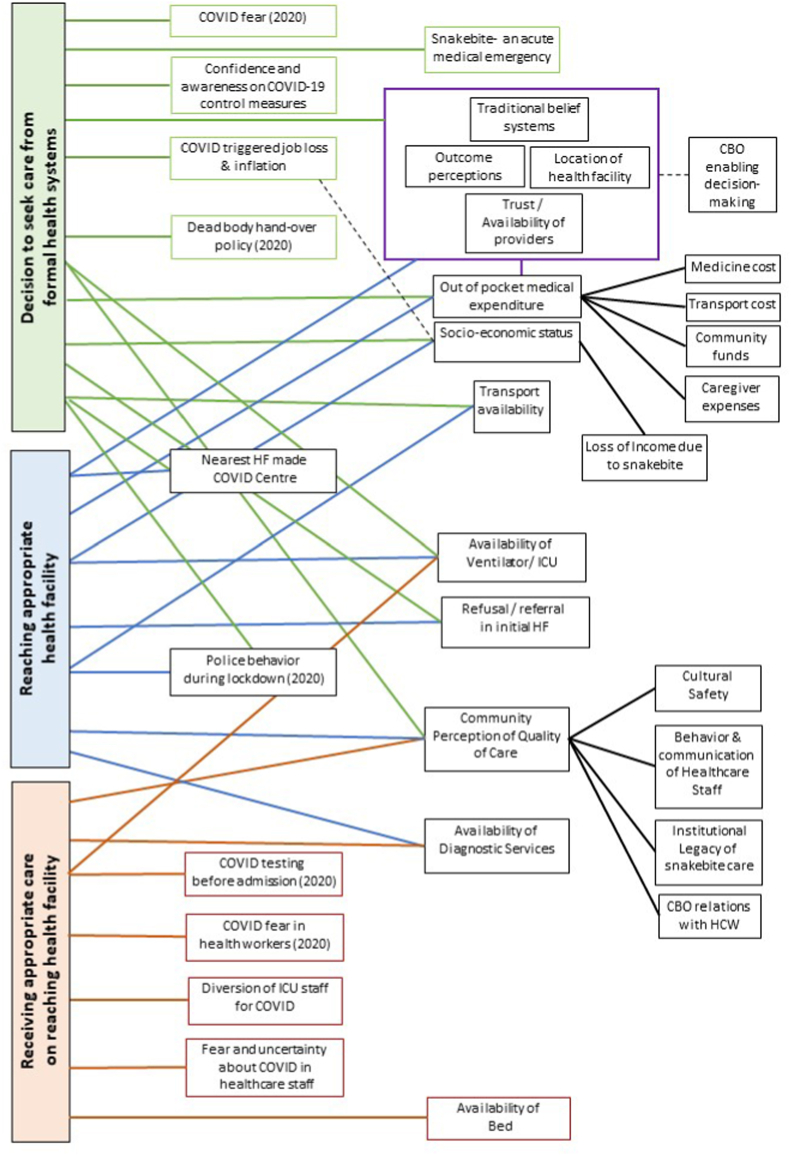
Factors affecting snakebite care mapped in three-delay framework

All factors which affect snakebite care are mapped diagrammatically in the three delay model (originally developed for maternal mortality (Thaddeus and Maine, 1994) in Fig. 2. The mapping is done to enhance visibility and recognition of the entire gamut of factors at play through not only the two waves of COVID-19, but also irrespective of the pandemic. The factors map to three levels (often multiple) and are related to.

• decision to seek care from formal health system,
• reaching an appropriate health facility, and
• receiving appropriate care after reaching a health facility.

While some factors are pre-existing, some are specific to COVID-19.

While the mapping in “three delays model” illustrates all factors affecting snakebite care, the themes identified in the primary analysis (i.e., thematic analysis) add texture and details beyond mapping in different aspects of care. In relation to the themes from our primary analysis the theme of snakebite being recognised as an acute medical emergency (theme 1) through successive waves of COVID-19, maps only to the first aspect in the three-delay model. On the other hand, the issue of financial woes of COVID-19, and the multifactorial nature of healthcare provider choice (themes 2 and 3) maps to the first and second aspects of the three-delay model. The issue of rurality, financial and social disadvantage (theme 4) maps to the second and third aspect of the three-delay model. The felt need of community-based program (theme 5) maps to all three aspects of the three-delay model.

## 4 Discussion

The result of our study contextualises and brings forth evidence with respect to impact of COVID-19 on snakebite care in West Bengal, India. Our study found that communities affected by snakebite are immensely challenged by weak health systems which was accentuated during the pandemic. Snakebite was recognised as an acute medical emergency and people navigated a multitude of factors which affected access to snakebite care, including distance, availability, trust, outcome perceptions, and affordability of formal health systems. We found that these factors (which accentuated during COVID-19), and not traditional belief systems alone, influenced the choice of healthcare provider. COVID-19 added to the financial risk of communities affected by snakebite. The lack of cultural safety and respectful care contributed to perceptions of poor quality of care. There is a strong and shared felt need for multi-faceted community-based programs on snakebite. However, we found that CHWs, saw a limited role in such a program. In contrast CBOs, where they existed, were recognised and appreciated by communities. CBOs were, however, challenged by lack of recognition and resource constraints.

The previous global qualitative study (van Oirschot et al., 2021) relied on key-informants alone and focussed on the initial phase of COVID-19. Our study was localised within a sub-national context and had community level participants, enabling us to look at the issue in more depth. Having two contrasting study areas within a state, also enabled comparison. The optimism of greater availability of ventilators for snakebite patients in the previous study (van Oirschot et al., 2021), is not reflected in our study. Our study on the other hand highlights the need for a simultaneous strengthening of primary health care systems and multi-faceted community-based programs to address snakebite and snake-human conflicts. The finding that CHWs did not see any major role in relation to snakebite, in the background of overwork, stress, and their identity, is in alignment with what has been seen in other studies on CHWs in India (Aryal & D'Mello M, 2020; Bhaumik et al., 2020; Dhaliwal et al., 2021; George et al., 2017). COVID-19 has exacerbated the issue, adding to concerns about financial security, occupational health and safety and psychosocial stress, leading to increasing collective action with some state governments around labour rights (Aryal & D'Mello M, 2020; Bhaumik et al., 2020; Dhaliwal et al., 2021; George et al., 2017). The current qualitative study, conducted in a single state of India, also adds to our regression analysis of facility-level data from multiple states, which estimated decrease in envenoming snakebite admissions, when health facilities were within a COVID containment zone, compared to when they were not, in the first wave of COVID-19 (Bhaumik et al., 2023). Among other things the quantitative study identified the gap in literature to understand factors affecting access to snakebite care – this qualitative study contributes towards filling this gap.

Difficulties in accessing care for multiple conditions due to COVID-19, has been noted in other studies from India, as well as globally (Kaushik et al., 2021; Keshri et al., 2021; Laura et al., 2021; Lipman et al., 2021; Prajitha et al., 2021; Raman et al., 2021; Rathore et al., 2021; Tandon, 2020). With the majority of global deaths due to snakebite occurring in India (Suraweera et al., 2020; WHO, 2019), action to reduce the burden in India is a priority to meeting the global reduction target. Our study provides a nuanced understanding, away from the dominant dichotomous framing (traditional belief systems versus modern medicine) (Bawaskar et al., 2020; Bhaumik, 2013; Chakma et al., 2020; Menon et al., 2017) around choice of healthcare providers for snakebite. The multi-factorial nature of decision-making to choose healthcare provider for snakebite has also been previously reported in Cameroon and Kenya (Chuat et al., 2021; Wood et al., 2022).

The novelty of our study also lies in mapping all the factors which affect snakebite care in the three-delay model (Fig. 2). Framing and documentation of factors affecting snakebite care has not been done previously. The three-delay visualisation functions as a multi-dimensional tool which district program managers and policy makers can use (together with health workers, communities, and other stakeholders) to identify system-level bottlenecks. Used prospectively, it allows anticipation of drawbacks for which counteractive strategies are required for. It can also be used retrospectively for auditing and reviewing deaths. Death audits and reviews might be community-based, facility-based or a combination of both (confidential enquiry) (Willcox et al., 2020). For all types, our visualisation helps establish a “social diagnosis” of avoidable factors which contribute to serious outcomes. A previous study conducted in India found that death audit and reviews for neonatal, infant and under-five deaths, conducted in a participatory manner and using the delays framework, led to recognition of factors and subsequent collective action to address deaths (Nandan et al., 2005). Our work lays the foundation for conduct of similar work on snakebite. Our study is conducted in West Bengal but might have relevance beyond West Bengal - in similar contexts of high snakebite burden and under-resourced health systems. Potential implications for policy, practice and research are presented in Table 2.

**Table 2:** Implications for practice, policy, and research.

1. Well-resourced multi-faceted community programs, involving local CBOs, have the potential to address factors which affect snakebite care, including during disease outbreaks, thus improving health systems resilience. Well-resourced community-based programs which aim for awareness, prevention (using contextually relevant modes and medium, as for example *Jatras* in West Bengal), increasing use of formal health services, and mitigation (snake-rescue) of snake-human conflict.
2. Community-based programs aiming to increase use of formal health services should be accompanied by health systems strengthening instead of an exclusive focus on awareness against traditional providers, with the underlying assumption that their acceptability is solely due to traditional belief systems.
3. Training for doctors and nurses for in-facility management of snakebite should include training on culturally appropriate and empathetic patient communication. Such training will reap benefits across all health conditions.
4. Future studies are needed to comprehensively map care pathways using mixed-methods approach to better understand care-seeking and navigation pathways from the point a person is bitten by snake to till death, recovery, or rehabilitation. This will also provide quantitative estimate of delays and, enable understanding of how delays affect outcomes.
5. Studies to understand out-of-pocket expenditure and its components are urgently needed. This can inform development of unconditional direct benefit transfer (DBT) schemes to enable protection of those affected by snakebite. The DBT scheme for tuberculosis has been found to be beneficial. (Dave and Rupani, 2022)
6. High burden states should commission district level evaluation of emergency response services to inform district level plans for ensuring adequate density and dispersion of ambulances, which are free and available 24*7.
7. Good practices from primary care facilities should be formally documented and scaled up.
8. In Sundarbans, and other hard to reach areas, surrounded by waterways, studies are needed for appropriate localisation of primary health centres and development of ferry-based emergency response. Geographic Information System based studies on snakebite epidemiology for the purpose have been conducted in other countries (Hansson et al., 2013; Molesworth et al., 2003) and are underway in Hooghly, West Bengal. (Malhotra et al., 2021)

We used standard qualitative research methods and reached saturation of themes. We acknowledge the limitation that we did not specifically explore how COVID-19 vaccination status affected risk perceptions to access snakebite care in formal health systems. The decision of diagrammatic presentation using three delay model (Thaddeus and Maine, 1994) was *post hoc*, and from a pragmatic standpoint of visualisation (rather than a descriptive list of factors). The three-delay model, is widely known and understood in communities of public health practice and policies. Our data effortlessly fitted within the model. It is envisaged that the figure will enable systems managers and policy actors to visualise factors related to snakebite care.

## 5 Conclusion

Well-resourced multi-faceted community programs, involving local CBOs, have the potential to address factors which affect snakebite care, including during disease outbreaks. Community-based programs aiming to increase use of formal health services should be accompanied by health systems strengthening (focussing on access, quality, cultural safety in practice and resilience) instead of an exclusive focus on awareness against traditional providers.

## Credit author statement

Soumyadeep Bhaumik - Conceptualisation, Funding acquisition, Methodology, Data-collection, Validation, Formal analysis, Writing – original draft, Writing – review & editing. Deepti Beri- Data-collection, Formal analysis, Writing – review & editing. Jagnoor Jagnoor- Methodology, Supervision, Writing – review & editing. Anthony B. Zwi – Conceptualisation, Supervision, Writing – review & editing.

## Ethical considerations

Ethics approval was obtained from The George Institute of Global Health, India (09/2020), and University of New South Wales (UNSW) Research Ethics Committee (HC220177). We provided a participant information sheet (in Bangla) and supplemented this with verbal explanations. Written informed consent was obtained from all participants.

## Funding

This work was supported by 10.13039/501100000683Royal Society of Tropical Medicine and Hygiene. The funders had no role in study design, data collection and analysis, decision to publish, or preparation of the manuscript.

## Declaration of competing interest

The authors declare that they have no known competing financial interests or personal relationships that could have appeared to influence the work reported in this paper.

## Data Availability

The data that has been used is confidential.

## Footnotes

1 In Sundarban, the Government of West Bengal provided relief for COVID-19 and co-incident Cyclone Amphan in May 2020 and Cyclone Yaas in May 2021.
